# Experimental and Numerical Study of Vacuum Resin Infusion of Stiffened Carbon Fiber Reinforced Panels

**DOI:** 10.3390/ma13214800

**Published:** 2020-10-28

**Authors:** Francesca Lionetto, Anna Moscatello, Giuseppe Totaro, Marco Raffone, Alfonso Maffezzoli

**Affiliations:** 1 Department of Engineering for Innovation, University of Salento, Via Arnesano, 73100 Lecce, Italy; anna.moscatello@alpak.it (A.M.); alfonso.maffezzoli@unisalento.it (A.M.); 2 Leonardo S.p.A., Divisione Velivoli, Viale dell’Aeronautica snc, 80038 Napoli, Italy; giuseppe.totaro02@leonardocompany.com (G.T.); marco.raffone@leonardocompany.com (M.R.)

**Keywords:** vacuum resin infusion, permeability, curing kinetics, chemo rheology, composite manufacturing, numerical simulation

## Abstract

Liquid resin infusion processes are becoming attractive for aeronautic applications as an alternative to conventional autoclave-based processes. They still present several challenges, which can be faced only with an accurate simulation able to optimize the process parameters and to replace traditional time-consuming trial-and-error procedures. This paper presents an experimentally validated model to simulate the resin infusion process of an aeronautical component by accounting for the anisotropic permeability of the reinforcement and the chemophysical and rheological changes in the crosslinking resin. The input parameters of the model have been experimentally determined. The experimental work has been devoted to the study of the curing kinetics and chemorheological behavior of the thermosetting epoxy matrix and to the determination of both the in-plane and out-of-plane permeability of two carbon fiber preforms using an ultrasonic-based method, recently developed by the authors. The numerical simulation of the resin infusion process involved the modeling of the resin flow through the reinforcement, the heat exchange in the part and within the mold, and the crosslinking reaction of the resin. The time necessary to fill the component has been measured by an optical fiber-based equipment and compared with the simulation results.

## 1. Introduction

Fiber-reinforced plastics (FRPs) are experiencing widespread use in the aeronautical, automotive, marine, energy, and construction field where the need for materials that are stiff and light at the same time is constantly growing [1,2,3,4,5]. The research in composite materials is very active both on the development of multifunctional, nanostructured, or environmentally friendly composite materials and on the technological challenges in fabrication and characterization [6]. Since the production of large, complex, and high-performance FRP structures at low costs is challenging, in recent years, a family of processes, named liquid composite molding (LCM), progressed toward new solutions with the aim of reducing costs and cycle times, thus becoming attractive for aeronautic applications as an alternative to conventional autoclave-based processes [7,8]. In LCM processes, prepregs are replaced by dry fiber preforms, i.e., an assembly of dry fibers plies that have been pre-shaped to the form of the desired product [9,10], which must be impregnated with resin, either via resin transfer molding (RTM), at pressure higher than 1 bar, or liquid resin infusion (LRI), just under vacuum [11,12]. LRI processes are characterized by several advantages such as the fabrication of large assemblies in one-shot, the substantial reduction in tooling costs, the minimization of mold and laminate deformation during the process, and the reduced emission of volatile components into the workplace [13].

Process challenges, such as consistent fiber impregnation, low porosity, and reduced filling time, can be faced only with a correct design of the process which is based on the analysis of resin flow during the infusion process [14]. Numerical simulation is a powerful tool for the design and optimization of the process parameters, enabling the replacement of traditional time-consuming trial-and-error procedures [15], prediction of resin flow path and infusion times, definition of the process conditions, and optimization of inlet ports and vents in order to minimize porosity [16,17]. Recently, some models have been developed for resin flow in LCM processes but they often do not consider industrial conditions and the high computational costs and long simulation times do not match the industry requirements [18,19]. Very few works are extended to investigate through-thickness flows during infusion [20,21], the main mechanism of impregnation in LRI.

Even though a few commercially available software packages have been developed, the simulation setting and computation time are very long, mainly with large and complex parts, the most attractive for the application of LRI to fabricate aeronautic parts. Therefore, it is very important to reduce the simulation time to obtain the best process design in the shortest period of time [22].

Moreover, for a realistic simulation of an industrial process, several experimental parameters, such as the thermal-kinetic and rheological properties of the resin, should be properly measured and given as an input [23]. During LRI, the thermosetting resin undergoes a chemical crosslinking reaction, named curing, which implies a change from the viscous liquid state to a gelled state and, finally, to a glassy solid state. Therefore, during infusion, the resin should have the time for impregnating and saturating the reinforcing preform, filling the porosities, and promoting an intimate contact with the fibers before the gelation, after which resin flow is no longer possible [19].

Moreover, the success of the resin infusion process relies on a proper choice of process parameters such as the positioning of inlet and outlet points, the temperature, the vacuum level, and the permeability of the preform, which characterizes the ability of a viscous liquid to impregnate a porous medium [24,25]. The permeability is of fundamental importance and strongly depends on the type of reinforcement, fiber orientation, and preform architecture when multi-layered preforms with different reinforcement types, e.g., unidirectional non-crimped fabrics and woven fabrics, are used to achieve the required mechanical performances [16]. Since permeability of a reinforcement preform is usually anisotropic, the resin does not flow at the same velocity in warp or weft direction and between in-plane and through thickness directions, where it flows very slowly.

In order to reduce the infusion time and promote the resin flow before gelation, often a highly permeable distribution medium (DM) is placed at the top or the bottom of the preform. Since the difference of permeability between the DM and the preform is very high, the resin flows in-plane first through the distribution medium and later within the preform following an out-of-plane direction with a resultant three-dimensional flow [26]. For this reason, the through-thickness flow plays an important role in the mold filling process of large and geometrically complex composite laminates. An accurate characterization of the in-plane and out-of-plane permeability is, therefore, fundamental to estimate the optimum process parameters for manufacturing high-quality components. Unfortunately, the permeability data are not available from the fabric manufacturer and must be measured depending on the fiber volume fraction and of the preform architecture. Often, a multi-layer fiber preform is used, composed of several different layers whose orientation and stacking sequence should optimize the mechanical performance. Different measurement methods are available in the literature, mostly related to the in-plane permeability, but there is not yet a standard procedure [27,28] and the data, obtained using different methods and different fabric architecture, are often not consistent [29]. Very few studies are reported on out-of-plane permeability, which is more difficult to measure [30,31].

This paper presents an experimentally validated model to simulate the resin infusion process of an aeronautical component by accounting for the anisotropic permeability of the reinforcement and the chemophysical and rheological changes in the crosslinking resin. The input parameters of the model have been experimentally determined. The experimental work has been devoted to the study of the curing kinetics and chemorheological behavior of the thermosetting epoxy matrix and to the determination of both in-plane and out-of-plane permeability of two carbon fiber preforms using an ultrasonic-based method recently developed by the authors. The numerical simulation of the resin infusion process involved the modeling of the resin flow through the reinforcement, the heat exchange in the part and within the mold, and the crosslinking reaction of the resin. The time necessary to fill the component has been measured by an optical fiber-based equipment and compared with the simulation results.

## 2. Mathematical Models for Numerical Simulation

The numerical simulation of the resin infusion process involves the modeling of the resin flow through the reinforcement and the crosslinking reaction of the resin [8]. The resin flow through the fiber reinforcement is modeled by Darcy’s law [32]:

(1) $$\vec{u}=-\frac{\tilde{K}}{\eta }\nabla P$$

where $\vec{u}$ is the resin velocity at the flow front, $\tilde{K}$ the permeability tensor of the reinforcement, *η* the resin viscosity, and ∇*P* the pressure gradient in the liquid phase. Darcy’s law for the resin flow can be integrated into the mass conservation equation for incompressible fluids:

(2) $$-\nabla \times \vec{u}=-\nabla \times \left(-\frac{\tilde{K}}{\eta }\nabla P\right)=0$$

The crosslinking reaction can be modeled by kinetics equations, among which Kamal’s equation [33,34,35] is one of the most used, relating the rate of curing reaction ∂*α*/∂*t* to the degree of curing α of the resin:

(3) $$\frac{\partial \alpha }{\partial t}=(k_{c1}+k_{c2}\alpha^{m}){\left(1-\alpha \right)}^{n}$$

where *m* and *n* are the reaction orders, while *k*_c1_ and *k*_c2_ are the kinetic constants.

Due to the chemical reaction of the resin, its viscosity *η* changes, being a function of the temperature *T* and degree of curing α:

*η* = *f*(α, *T*) (4)

A chemorheological model, able to fit the evolution of viscosity and degree of curing with the temperature, has been implemented in the flow front simulation in order to obtain more realistic filling simulations as will be better explained in the following paragraphs.

## 3. Experimental

### 3.1. Component Geometry and Reinforcement

**Figure 1:**
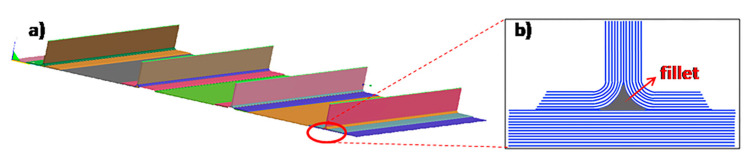
(**a**) Stiffened panel; (**b**) front view of the bottom of the stringer in contact with the flat laminate.

The studied component, representative of an aeronautic structural element, is a flat laminate stiffened by four stringers (Figure 1a). The lay-up of the laminate (813 × 559 × 2.4 mm^3^) was [45/90/0/±45]^2^ and that of the stringers (813 × 88.5 × 38 mm^3^) was [45/−45/0/90/0/−45/45]. Both the flat laminate and the stringers were made by resin infusion in intermediate modulus carbon fiber preforms obtained by a unidirectional TX 1100 IMS65 24k fabric (Solvay S.A., Bruxelles, Belgium), prepared by automated fiber placement (AFP) of unidirectional tapes. The fillet of the stringer, evidenced in Figure 1b, was made of BNFC-24k IMS-(0)-196-600 (Solvay S.A., Bruxelles, Belgium) fabric with 0° orientation. The nominal thickness of both carbon fiber tapes was 0.2 mm, the areal weight was 200 g/m^2^, and the elastic modulus was 290 GPa, as reported in the technical datasheet.

The thermosetting matrix used for the infusion process was PRISM^®^EP2400, produced by Solvay S.A. (Bruxelles, Belgium). It is a one-part toughened injectable epoxy resin, developed for the infusion of primary aeronautic components, able to provide increased toughness and improved processability. According to the technical datasheet, the injection temperature should be between 90 and 120 °C. The cure cycle recommended by the manufacturer consists of heating at 2 °C/min until 180 °C and a hold time of 180–210 min at 180 °C, able to provide a resin for a service temperature above 120 °C [36].

The infusion process was performed adopting a resin distribution medium, Resinflow 90 HT, specifically designed for resins that cure at 180 °C, and the Securlon L-500Y vacuum bagging film, both produced by Airtech Europe Sarl (Differdange, Luxembourg). In order to limit the increase in viscosity due to the reaction and to keep the viscosity adequately low, the vacuum infusion process was carried out at 100 °C in a heated oven under standard industrial conditions in the Leonardo S.p.A factory in Foggia (Italy). Before infusion, both the resin and the carbon fiber preform were preheated at the infusion temperature for 2 h, as recommended by internal industrial protocols.

### 3.2. In-Plane Permeability Measurement

**Figure 2:**
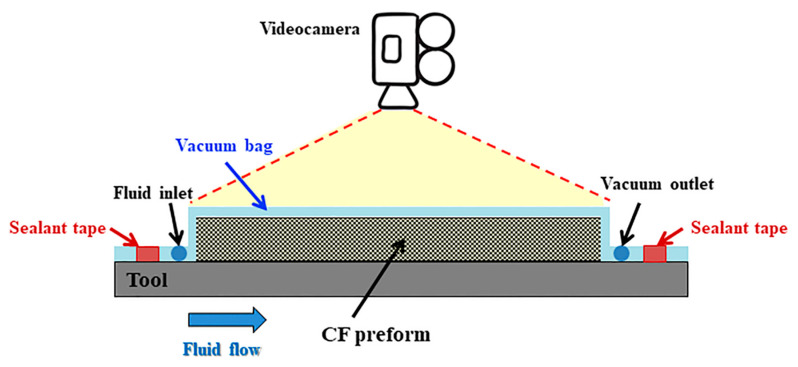
Sketch not in scale for the measurement of the in-plane permeability in a resin infusion process.

In-plane permeability measurements of the carbon fiber preforms and the distribution medium were carried out by the unidirectional flow method at constant injection pressure [37,38,39]. Both carbon fiber (CF) preforms (70 × 60 × 8 mm^3^) and distribution medium specimens (70 × 60 × 0.6 mm^3^) were tested. As sketched in Figure 2, the dry carbon fiber preform (or the distribution medium) was placed on a steel tool and covered by a vacuum bag film (Securlon L-500Y produced by Airtech Europe Sarl, Differdange, Luxembourg). A silicone seal was used to avoid any leakage during the measurement. The vacuum line was connected to a resin trap, a vacuum pump, and a pressure transducer.

Polyethylene glycol 400 (PEG400), provided by Sigma-Aldrich (Milano, Italy), was used as a test fluid. Its viscosity was measured at 25 °C in a parallel plate ARES rheometer (Rheometric Scientific, Piscataway, NJ, USA) with a 50 mm plate diameter in order to obtain a fluid at room temperature characterized by the same viscosity of the PRISM resin at the infusion temperatures, which is in the range of 0.06–0.1 Pa·s [28]. The measurement of in-plane permeability was performed during a Vacuum-Assisted Resin Infusion (VARI) process. When the vacuum was applied, the fluid was drawn into the mold by vacuum, thus impregnating the preform according to a unidirectional flow. Particular attention was paid to avoid any race-tracking effect. During the VARI process, the transparent vacuum bag film enabled the monitoring of the front flow position, which was recorded by a video camera, as sketched in Figure 2. A ruler with ticks at each mm was adopted to measure the instantaneous front position. The in-plane permeability values *K*_1_ and *K*_2_ were determined using Darcy’s law, as explained later in paragraph 4.1. Three preforms of each carbon fabric (TX1100 and BNCF) were analyzed.

### 3.3. Out of Plane Permeability Measurement

**Figure 3:**
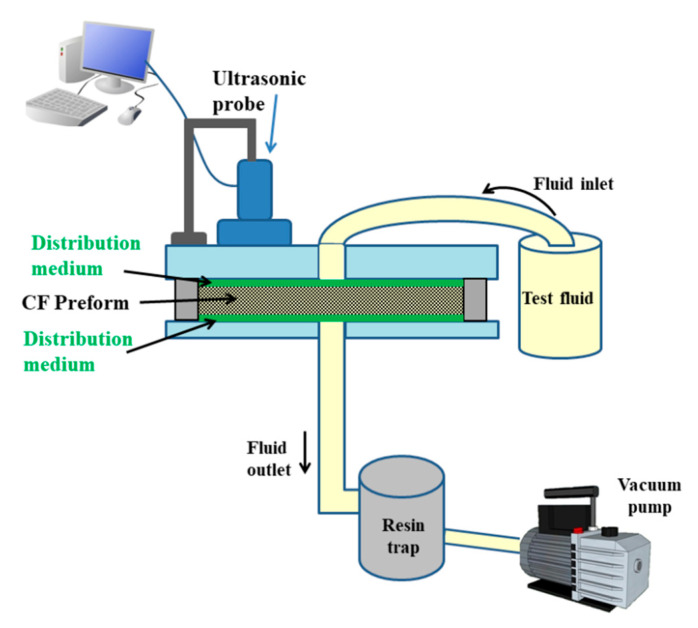
Experimental setup not in scale for transverse permeability measurements by ultrasonic wave propagation.

The experimental setup for the measurement of out of plane, non-saturated, permeability by ultrasonic wave propagation in pulse echo mode is sketched in Figure 3 [28]. A single ultrasonic transducer, working both as emitter and receiver of ultrasonic waves, was used to monitor the through thickness flow front during a vacuum-assisted resin infusion experiment. The out of plane permeability was also determined applying Darcy’s law in the same configuration as Figure 3, adopting a steady state flow, i.e., in saturated conditions, as reported in a previous study [28].

### 3.4. Resin Characterization

The cure kinetics of the resin was studied by differential scanning calorimetry (DSC) using a DSC 822 calorimeter (Mettler Toledo, Greifensee, Switzerland). Dynamic DSC scans were performed on uncured resin samples from 25 to 300 °C at constant heating rates (1, 2, 5, and 10 °C/min) in a nitrogen atmosphere. Three replicates for each heating rate were performed.

Rheological analysis was performed using a parallel plate rheometer (ARES, Rheometric Scientific, Piscataway, NJ, USA) with 50 mm plate diameter in dynamic mode at a frequency of 1 Hz during a temperature scan from 50 °C to, at maximum, 240 °C at 1, 2, and 5 °C/min. To prevent the damage of the torque transducer, the dynamic scan was stopped when the resin viscosity reached a value of 1000 Pa·s, which occurred at different temperatures depending on the heating rate. Three replicates for each heating rate were performed.

### 3.5. Infusion Monitoring by Fiber Bragg Grating Sensors

The infusion process was monitored by Fiber Bragg Grating (FBG) sensors (T20, Technica Optical Components LCC, Atlanta, GA, USA). The measurements were carried out during the resin infusion of the stiffened panel in the industrial plant of Leonardo S.p.A. in Foggia (Italy) using FBG sensors coated by a polyimide coating with an outer diameter of 155 μm and a Bragg wavelength of 1550 nm.

### 3.6. PAM-RTM

The 3D simulation of the resin filling of the stiffened panel was carried out using PAM-RTM 2015.0 software (Esi Group, Paris, France) [40]. Since the fiber orientation in each ply of the preform was different, a 3D flow simulation was carried out. The software used Darcy’s Equation (1), the mass conservation Equation (2), and Kamal’s Equation (3) for simulating the filling of the preform. Since the aim of the work was to simulate the filling in industrial conditions where usually the resin is heated in big tanks for a couple of hours before infusion, it is necessary to take into account the changes of resin viscosity due to slow curing at injection temperature. For this reason, in this work, a chemorehological model was implemented into the FEM software. The details of the model will be explained in the next paragraphs.

## 4. Results and Discussion

### 4.1. Permeability Measurement Results

**Figure 4:**
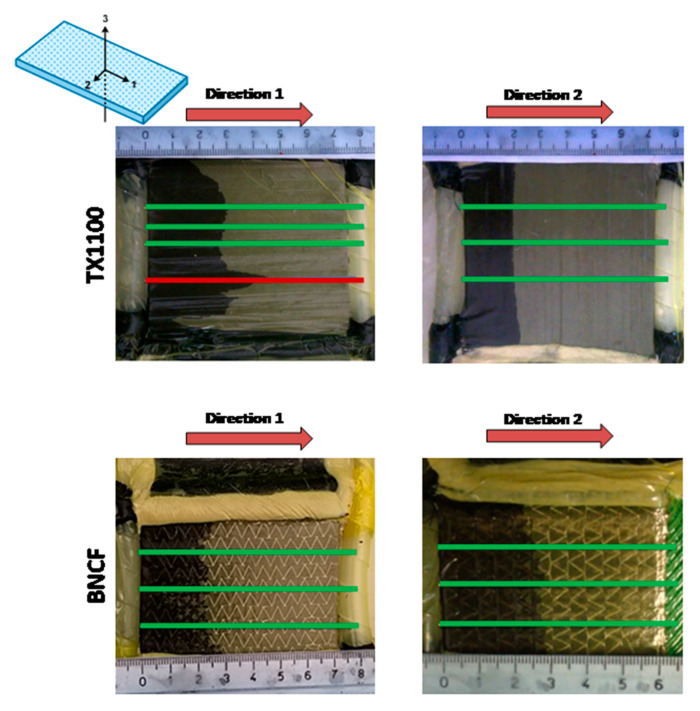
Flow front monitoring along the directions 1 and 2 for the two studied preforms.

Some frames of flow front monitoring for the in-plane permeability measurement are shown in Figure 4. The in-plane permeability measurements have been carried out along the directions 1 and 2 of a unidirectional preform, i.e., across in-plane directions. For each preform and flow direction, the flow front position has been obtained as the mean value among those measured along three different measurement lines, evidenced by green lines in Figure 4. As observable in Figure 4, the measurement lines have been chosen in correspondence of a homogenous flow front and far from the edge zones and the race tracking zones (as evidenced by the red line for the TX1100 preform).

**Figure 5:**
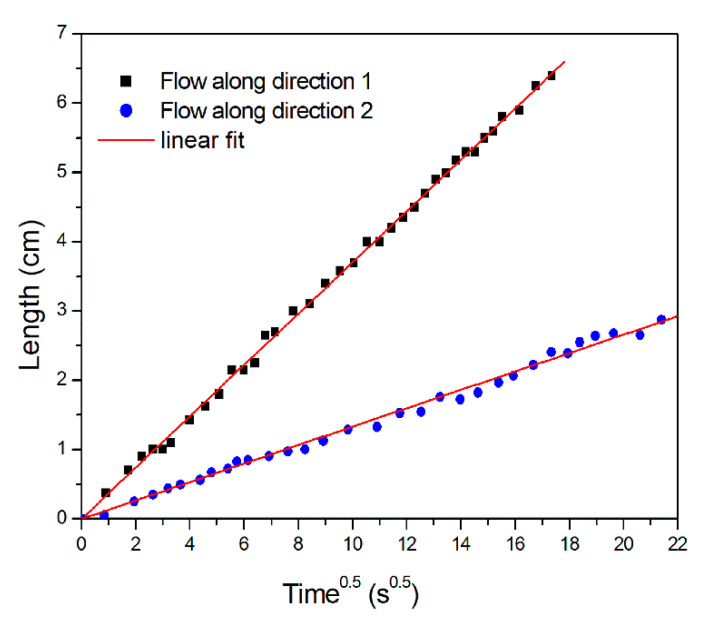
Flow front position along 1 and 2 direction as a function of the square root of time for TX 1100 preform.

The infusion has been videorecorded and the progress of the flow front position has been measured by a graduate scale at time steps of 5 s. By integrating Darcy’s equation for one-directional, constant pressure, and incompressible flow, the flow front position *x*_f_ is given by:

(5) $$x_{f}=\sqrt{\frac{2K\Delta P}{\eta (1-V_{f})}}\sqrt{t}$$

where *η* is the fluid viscosity, *V_f_* the fiber volume fraction, Δ*P* the pressure drop between the inlet and the flow front, and *t* the infusion time. A plot of the flow front position *x_f_* as a function of the square root of the time is characterized by a linear behavior, as shown in Figure 5, along the two directions 1 and 2. In-plane permeability *K_i_* along in-plane directions 1 and 2 is obtained from the slope of the linear fitting of data reported in Figure 5 as:

(6) $$K_{i}=\frac{slope\cdot \eta (1-V_{f})}{2\Delta P},\;\mathrm{where}\;i\;=\;1,\;2$$

The fiber volume fraction *V_f_* of the perform, necessary for *K* determination, can be obtained according to the following equation:

(7) $$V_{f}=\frac{nS_{0}}{\rho_{f}h}$$

where *n* is the number of plies, *S_0_* the areal weight of a single ply, *ρ_f_* is the fiber density, and *h* the preform thickness.

**Figure 6:**
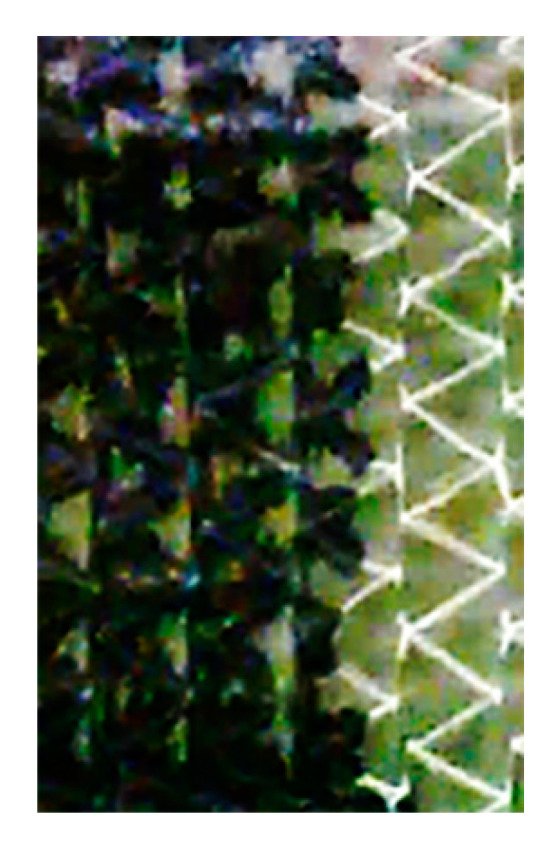
Flow front along stitches during infusion of BNCF preform.

The in-plane permeability values of the carbon fiber fabrics at the fiber volume content of 55% are reported in Table 1. As expected, the TX 1100 preform based on unidirectional tape shows a pronounced anisotropy in the permeability values. *K*_2_, measured along the direction perpendicular to the fibers, is of one order of magnitude lower than the *K*_1_. Contrary to what is expected, this difference does not exist in the case of the BNCF preform, where the *K* values along the in-plane directions 1 and 2 are comparable, although the preform is unidirectional. This is due to the presence in each ply of stitches, which become preferential flow patterns, as shown in Figure 6. The flow is promoted by stitches along a zig-zag path, while the empty triangular spaces between stitches are subsequently filled. Therefore, in the case of stitched fabric, the filling process of the fiber tows is a combination of 1D flow in the stitches, actually acting as resin distribution media, and 2D flow between them. The permeability has been measured using the average position observed after complete filling of each mesh formed by stitches.

The out of plane measurement of permeability by ultrasound has been detailed in a previous work [28]. The permeability values, used as an input for the simulation, in correspondence of fiber volume content of 55%, are reported in Table 1. It should be underlined that for the carbon fiber preforms, the *K*_3_ values are two orders of magnitude lower than the *K*_1_ values. For the distribution medium, only the in-plane permeability has been measured, considering its isotropic behavior.

### 4.2. Chemorheology of the Epoxy Matrix

**Figure 7:**
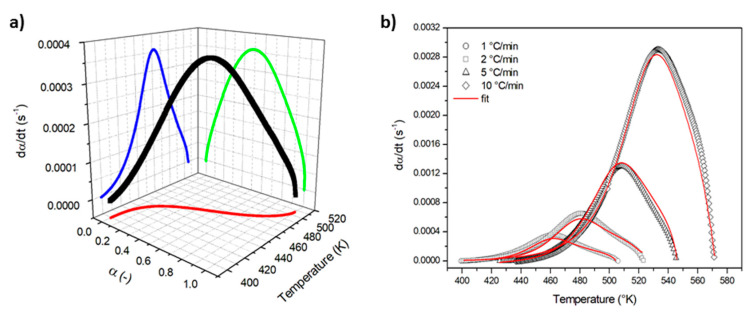
**(a**) 3D plot of the reaction rate *dα/dt* at 1 °C/min as a function of the temperature and the degree of reaction *α*; (**b**) comparison of experimental reaction rate and Equation (8) predictions.

The kinetic analysis of the curing process has been performed by dynamic DSC scans on the uncured epoxy matrix at the typical heating rates adopted during autoclave curing. By assuming that the heat flow measured in a DSC experiment is proportional to the rate of the exothermic crosslinking reaction, the reaction rate *dα/dt* and the degree of reaction *α* have been determined [23]. As an example, a 3D plot of the reaction rate *dα/dt* at 1 °C/min as a function of the temperature and the degree of reaction α is reported in Figure 7a. The reaction rate *dα/dt* of the thermosetting matrix has been modeled by Kamal’s model [33,34,35,41], reported in Equation (3), taking into account that the rate constants *k*_c1_ and *k*_c2_ have an Arrhenius dependence on the temperature:

(8) $$\frac{d\alpha }{dt}=\left(k_{01}\exp\left(-\frac{E_{a1}}{RT}\right)+k_{02}\exp\left(-\frac{E_{a2}}{RT}\right)\alpha^{m}\right){\left(1-\alpha \right)}^{n}$$

where *k*_01_ and *k*_02_ are the reaction rate constants, *E*_a1_ and *E*_a2_ the activation energies, *R* the universal gas constant, and *m* and *n* the reaction orders. The kinetic parameters, obtained by Levenberg–Marquardt least-squares minimization, are reported in Table 2. The experimental reaction rates, as a function of the temperature and model predictions (full line), are compared in Figure 7b. At all the investigated heating rates, a very good correspondence between the non-linear fit and the experimental results over the whole range of curing temperatures was obtained.

**Figure 8:**
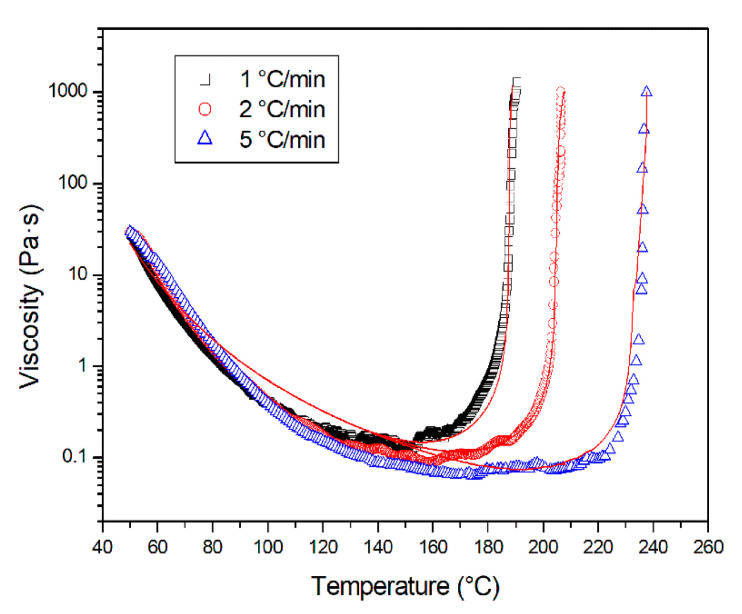
Viscosity plots for the PRISM EP 2400 resin at different heating rates. The red line represents the fitting according to the modified Kenny and Opalicki model.

The rheological analysis has been carried out since the viscosity of the resin, a key parameter for the correct infusion of the panel, depends on the temperature and the degree of reaction of the resin. The rheological curves at different heating rates are reported in Figure 8. Two competing phenomena can easily be observed: an initial viscosity reduction with increasing temperature, due to the increased molecular mobility, and, at higher temperatures with the advancing of the curing reaction, a viscosity increase due to the growth of molecular weight, until gelation is reached.

The chemorheological model, adopted to study the evolution of viscosity as a function of temperature and the degree of reaction, is a modified version of the Kenny and Opalicki model, given by the product of a function of the temperature and a function of the degree of reaction:

(9) $$\eta =\eta_{g0}\exp\left(\frac{-C_{1}(T-T_{g0})}{C_{2}+T-T_{g0}}\right){\left(\frac{\alpha_{g}}{\alpha_{g}-\alpha }\right)}^{A+B\alpha }$$

where *η*_g0_ is the viscosity of the unreacted resin at the initial glass transition temperature *T*_g0_, α_g_ is the degree of reaction at the gel temperature, while *C*_1_, *C*_2_, *A*, and *B* are the model parameters (Table 3). The degree of reaction α used in the chemorheological model has been obtained from the DSC analysis. The comparison among experimental values and model fitting, shown in Figure 8, is very satisfactory. The measured α_g_ value is equal to 0.46, i.e., it is in the typical 0.4–0.5 range expected for the epoxy resins [42].

### 4.3. Simulation of the Infusion Process of a Stiffened Panel

The simulation of the preform filling of the stiffened panel has been preceded by preliminary simulations on small components of simple rectangular geometry. These preliminary simulations were performed with the aim of validating both the experimental results of in-plane and out of plane permeability and proper setting of the initial and boundary conditions. Moreover, a sensitivity study has been carried out on the simplified geometry in order to choose the proper mesh size that provides adequate accuracy to the simulation [43].

**Figure 9:**
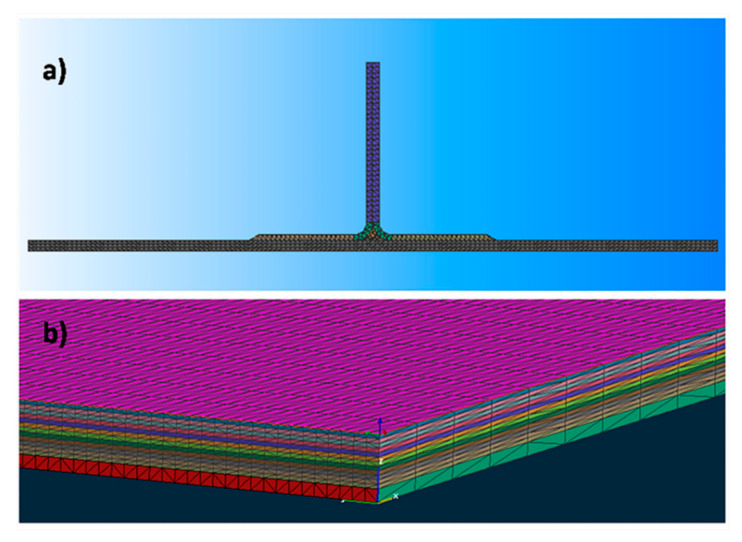
(**a**) Front view of the meshed component with the flat panel and the stringer, (**b**) meshed geometry of the base panel.

The model geometry used for the simulation is composed by the stiffened panel and a resin distribution medium placed at the bottom of the panel. In order to reduce the simulation time, a geometry smaller (100 × 559 mm^2^) than the actual geometry (813 × 559 mm^2^) has been simulated. The mesh size has been set to 0.6 × 4 × 0.6 mm^3^ for the resin distribution medium and 0.6 × 4 × 0.2 mm^3^ for the preform, i.e., the height of the mesh element corresponds to the thickness of each carbon fiber ply. A front view of the mesh is reported in Figure 9a, while in Figure 9b, a particular of the mesh adopted for the base panel is shown.

**Figure 10:**
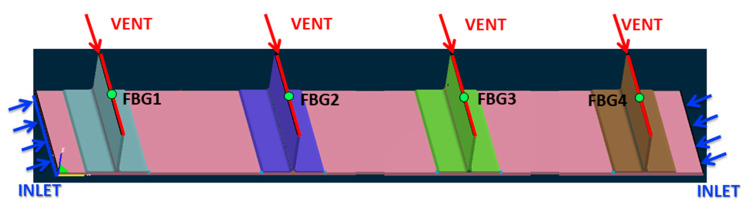
Resin inlets (blue arrows) and vents (red arrows) configuration.

The injection system is detailed in Figure 10. Two resin inlets, made of omega flow tubes, are placed symmetrically on the lateral sides of the base plate, as evidenced by the blue lines. Resin vents, evinced by red lines, are on the top of the stringers. The boundary conditions were ambient pressure at the inlet port and vacuum pressure at the vent port and ∂*P*/∂*n* = 0 at the mold and vacuum bag side. The simulation automatically stopped when all the elements were filled.

The effects of some technological parameters have been considered in the simulation. To this aim, a vacuum level of 0.9 and 0.98 bar and an injection temperature of 100 and 110 °C have been simulated. The choice of the isothermal filling temperatures has accounted for both the resin properties and the industrial conditions of the filling. According to the viscosity profile in the technical datasheet, the viscosity is very close to minimum already at 100 °C and its further decrement is small for temperatures higher than 110 °C, above which the resin reactivity cannot be neglected and a possible increase in viscosity associated with an increase in molecular weight is likely to occur. Moreover, the filling temperature must be selected in order to prevent any relevant increase in molecular weight of the epoxy resin in the tank, which is associated with an increase in viscosity not acceptable for preform filling under a very low pressure gradient. An incomplete filling of a large preform is expected at temperatures lower than 100 °C or higher than 110 °C, above which a relevant increase in viscosity, or even premature gelation of the resin, could occur.

**Figure 11:**
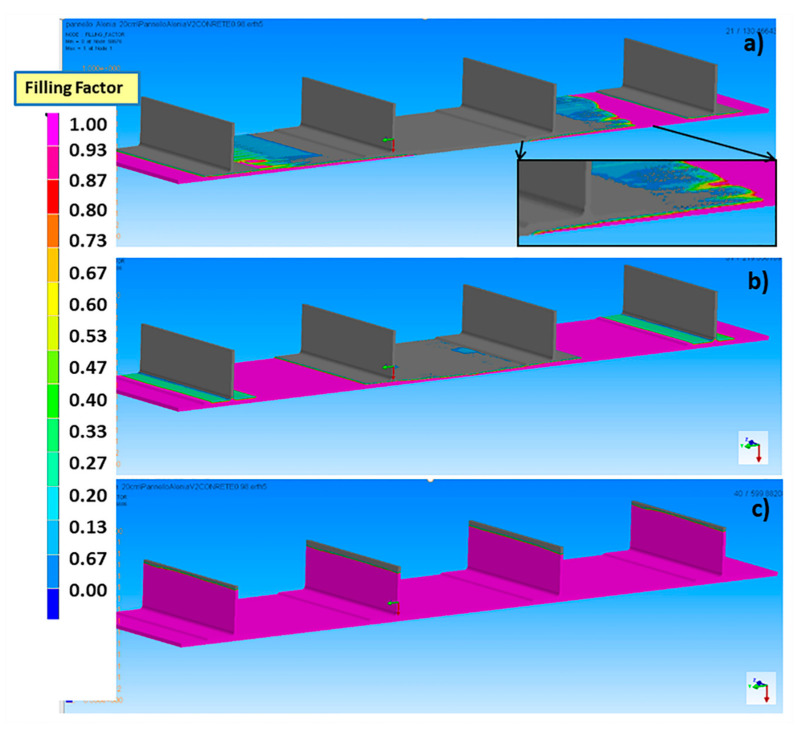
Different filling stages of the stiffened panel at 100 °C and 0.98 bar for different times: (**a**) 130 s, (**b**) 220 s, (**c**) 700 s.

The different filling stages of the stiffened panel are reported in Figure 11 for an infusion temperature of 100 °C and Δ*P* of 0.98 bar. The infusion of the panel, driven by the pressure difference between the inlet and the vent, proceeds symmetrically, starting from the two inlet ports on the lateral sides of the panel. The presence of the distribution medium at the bottom of the base panel, characterized by a significantly lower permeability than that of carbon fiber preform, enables at first the in-plane flow through the distribution medium and then, the out of plane flow through the thickness of the carbon fiber preform, as evidenced by the inset of Figure 11a. After filling the entire base laminate, the resin also continues to flow along the in-plane direction in the stringers, as shown in Figure 11b,c.

**Figure 12:**
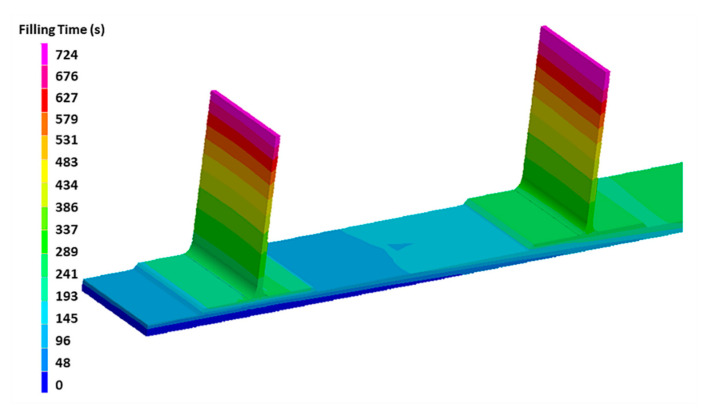
Filling time of the carbon fiber panel stiffened by stringers (half component is shown for clarity).

As an example of the simulation output, the filling time at 0.9 bar and 100 °C is reported in Figure 12, where only half the component is shown in order to clearly observe the differences. The base panel is filled after 340 s, while the top of the stringer is after 724 s. Since the stringers are filled with an in-plane resin flow, its filling is relatively faster than that of the base panel, where the flow advances mostly through the thickness, where the out of plane *K*_3_ permeability of the preform is about two orders of magnitude lower than the in-plane *K*_1_ permeability.

The effect of the vacuum level and filling temperature on the filling time is reported in Table 4. At the same filling temperature, a Δ*P* 10% higher corresponds to a reduction of about 8% in the filling time of the overall component. At the same filling pressure, an increase of 10 °C in the filling temperature, from 100 to 110 °C, leads to a decrease in the filling time of about 36%, as a consequence of a significant decrease in resin viscosity. Table 4 also reports the degree of curing α at the end of the filling stage. The latter has been determined using the finite element FlexPDE software (PDE Solutions Inc., Spokane Valley, WA, USA) to integrate the kinetic model given by Equation (8) in industrial conditions as those used in the factory during the infusion of the aeronautical components: a slow heating of the resin at 1 °C/min, an isothermal dwell stage of the resin at the filling temperature in the resin tank, and the isothermal filling of the preform. The temperature significantly affects the degree of curing at the end of filling stage, which results more than doubled, with an increase of 10 °C in the filling temperature.

**Figure 13:**
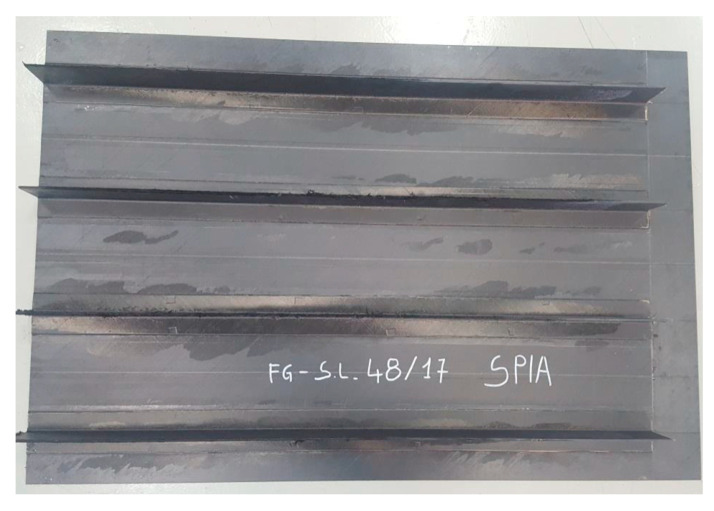
Top view of the stiffened panel manufactured by resin infusion.

During the design of the infusion process, two competing factors should be minimized: the filling time and the degree of curing. The first one has an effect on the cost of the process, while trying to limit the growth of the degree of reaction is needed to keep the viscosity low, with a consequently improved fiber impregnation and reduced void content. For this reason, the filling at 100 °C has been chosen for the resin infusion of the stiffened panel realized at the Leonardo S.p.A factory of Foggia (Italy), which is shown in Figure 13. A vacuum of 0.9 bar has been adopted, being the highest stable value that could be achieved in an industrial plant. This experiment in an industrial environment has been used as a validation of the simulation results of the filling time. The filling times have been measured by FBG sensors, located at the venting ports on the top of the stringers, as evidenced in Figure 10. The simulated times for the resin to reach the outlet at the top of the stringers is between 719 and 724 s, in very good agreement with the experimental data obtained from the optical fibers positioned at the same position on top of the stringers, as reported in Table 5. It should be underlined that an error occurred in the measurement of the FBG sensor located on the 4th stringer (FBG4). For this reason, the relative value is not reported in Table 5.

A very good agreement is observed between the simulated and the experimentally observed filling times, thus indicating that the input parameters and the governing equations are capable of providing reasonably good predictions.

## 5. Conclusions

This paper presents an experimentally validated model to simulate both the resin infusion and cure process under the industrial conditions of an aeronautical stiffened CFRP panel. The experimental work has been devoted to the determination of both the in-plane and out-of-plane permeability of two carbon fiber preforms used for the realization of the component, and to the study of the curing kinetics and chemorheological behavior of the thermosetting epoxy matrix. The effect of the stitching of hot bonded plies on the resin flow path was observed. The obtained permeability data were used as input for the simulation by finite element method of the filling of a panel stiffened by vertical stringers. DSC and rheological results enabled the set up of kinetic and chemorheological models that were coupled with the mass conservation and Darcy’s equation for simulation of the infusion process. The time necessary for the resin to fill the component was measured by an optical fiber-based equipment and compared with the simulation results. An optimum agreement between numerical and experimental filling results has been found that validates the proposed model.

## Figures and Tables

**Table 1:** Permeabilities of the carbon fiber fabrics.

Permeability	TX1100 Preform	BNCF Preform	Distribution Medium
*K*_1_ (µm^2^)	2.81 ± 0.55	1.73 ± 0.57	1700 ± 160
*K*_2_ (µm^2^)	0.38 ± 0.43	1.68 ± 0.01	1700 ± 160
*K*_3_ (µm^2^) [18]	0.043 ± 0.009	0.036 ± 0.009	not measured

**Table 2:** Kinetic parameters for the PRISM EP 2400 resin.

*k*_01_ (s^−1^)	*E*_a1_ (kJ/mol)	*k*_02_ (s^−1^)	*E*_a2_ (kJ/mol)	*m* (-)	*n* (-)
288,713	82.92	3596	60.43	0.45	0.89

**Table 3:** Chemorheological parameters for the PRISM EP 2400 resin.

*η*_g0_ (Pa·s)	*C*_1_ (-)	*C*_2_ (K)	*α*_g_ (-)	*A* (-)	*B* (-)
3.9 × 10^9^	28.7	30.7	0.46	2.05	1.9

**Table 4:** Effect of the vacuum level, filling temperature, and vent configuration on the filling time.

Filling Temperature (°C)	Δ*P* (bar)	Filling Time (s)	Degree of Curing α (%)
100	0.90	724	0.64
100	0.98	654	0.62
110	0.90	457	0.125
110	0.98	420	0.127

**Table 5:** Comparison between experimental and numerical results.

	Filling Time (s)	
	Numerical	Experimental
FBG1	719	764
FBG2	724	700
FBG3	724	705
FBG4	719	-
